# Presence of tophi and carotid plaque were risk factors of MACE in subclinical artherosclerosis patients with gout: a longitudinal cohort study

**DOI:** 10.3389/fimmu.2023.1151782

**Published:** 2023-04-18

**Authors:** Yu Wang, Xuerong Deng, Xiaohui Zhang, Yan Geng, Lanlan Ji, Zhibo Song, Zhuoli Zhang

**Affiliations:** Department of Rheumatology and Clinical Immunology, Peking University First Hospital, Beijing, China

**Keywords:** gout, tophi, crystal arthritis, cardiovascular disease, ultrasonography

## Abstract

**Background:**

Patients with gout carry an excess risk for cardiovascular disease (CVD), but the contribution of subclinical atherosclerosis to the CVD risk has never been reported. In this study, we aimed to explore the predictive factors for incident major adverse cardiovascular events (MACE) in gout patients without a previous history of CVD or cerebral vascular disease.

**Methods:**

A single-center, long-term follow-up cohort analysis was performed to assess subclinical atherosclerosis at baseline since 2008. Patients with a previous history of CVD or cerebrovascular disease were excluded. The outcome of the study was the first MACE. The presence of subclinical atherosclerosis was assessed by carotid plaque (CP), and carotid intima-media thickness (CMIT) was determined by ultrasound. An ultrasound scan of bilateral feet and ankles was performed at baseline. The association between tophi, carotid atherosclerosis, and the risk of developing incident MACE was evaluated using Cox proportional hazards models with adjustment for the CVD risk scores.

**Results:**

A total of 240 consecutive patients with primary gout were recruited. Their mean age was 44.0 years, with male predominance (238, 99.2%). During a median follow-up of 10.3 years, incident MACE was ascertained in 28 (11.7%) patients. In a Cox hazards model, controlling for the CV risk scores, the presence of at least two tophi (HR, 2.12–5.25, *p <* 0.05) and carotid plaque (HR, 3.72–4.01, *p <* 0.05) were identified as independent predictors of incident MACE in gout patients.

**Conclusions:**

The presence of at least two tophi and carotid plaque on an ultrasound could independently predict MACE in addition to conventional cardiovascular risk factors in gout patients.

## Introduction

Gout is a common chronic disorder resulting from the sustained elevation of serum urate acid (SUA) and gradual deposition of monosodium urate crystals in joints, tendons, and other tissues. Both the prevalence and incidence of gout seem to be rising globally, especially in developing countries (1). A meta-analysis of 44 studies published from 2000 to 2014 revealed that the pooled prevalence of gout was 1.1% in the adult Chinese population (2).

Gout is associated with a 17% increase in all-cause mortality risk, with cardiovascular disease (CVD) as the leading cause of death (3). A previous study found that patients with asymptomatic hyperuricemia and silent monosodium urate deposits suffered from a more severe form of coronary atherosclerosis (4). Atheroma plaque was observed by carotid ultrasound in 46.5% of gout patients with newly proven crystal deposition. Importantly, they were classified as having a very high risk of cardiovascular (CV) risk (5). To be noted, the cardiovascular risk in gout patients may be even higher than what has already been shown based on the standard evaluation. Moreover, the rationale for early and effective treatment of gout also greatly depends on the consideration of CV consequences.

Hyperuricemia is also closely associated with CVD, although whether this is due to covariation with the traditional risk factors or a causative role of its own has not been definitively elucidated. Hyperuricemia has also been identified as an independent risk factor for hypertension and impaired kidney function (6). A recent study showed that SUA level was associated with the coronary artery calcification score in male patients but not with carotid intima-media thickness (cIMT) or carotid plaque score (7). Of note, psoriasis is often associated with elevated serum uric acid levels. In this regard, in a cohort of patients with psoriatic arthritis without clinically evident cardiovascular disease, a correlation was found between the serum uric acid concentration and subclinical atherosclerosis measured by the thickness of the carotid intima-media (8).

Early recognition and intervention with risk factors for CVD may be beneficial in reducing CV events in patients with gout. Existing tools, such as the Framingham risk score (FRS), QRISK3, or Systematic Coronary Risk Evaluation (SCORE), have been validated in general population-based large cohorts with long follow-ups (9). It is noted that all these risk scores are based on the traditional CVD parameters, which may underestimate the real CVD risk in patients with rheumatic diseases. Therefore, EULAR recommends a 1.5 multiplication factor for the risk scores of patients with rheumatoid arthritis (10). In 2022, EULAR also recommended using these cardiovascular prediction tools for patients with gout. However, no study has been conducted to investigate the accuracy of these tools, and it is unclear to what extent the elevated risk for cardiovascular disease in gout patients is caused by an increased prevalence of traditional or disease-specific risk factors.

To address these important questions, we conducted this 10-year prospective study. The aims of the study were (1) to investigate whether subclinical atherosclerosis evaluated by carotid ultrasound could predict incident MACE in patients with gout; (2) to identify whether crystal deposition evaluated by ultrasound could improve the prediction of MACE and whether it is positively adjudicated by traditional clinical cardiovascular algorithms (11).

## Methods

### Study design and population

This study is based on a prospective cohort of gout patients established in the Department of Rheumatology and Clinical Immunology, Peking University First Hospital (PKU-GOUT) in 2008. Patients in the cohort are followed up at regular intervals, and the treatment decisions at each visit are made at the discretion of the attending rheumatologist based on assessment.

In this study, we enrolled those patients from the PKU-GOUT cohort who satisfied the following inclusion criteria: (1) fulfilled the 2015 American College of Rheumatology/European League Against Rheumatism (ACR/EULAR) Classification Criteria or 1977 ACR criteria for gout (12); (2) >18 years old; (3) with available ultrasound data of carotid artery as well as urate acid deposition in bilateral feet and ankles at the first visit to our clinic; (4) being naïve to urate-lowering therapy (ULT) or with previous ULT treatment 3 months ago; and (5) experienced flare of gouty arthritis at least once within the last 3 months before enrollment.

Patients with any of the following conditions were further excluded: diagnosed stroke or CVD, including a history of myocardial infarction, coronary artery bypass graft surgery, or abnormalities on cardiac tests (exercise treadmill testing, echocardiography, myocardial perfusion, abnormal cardiac computed tomography, or coronary angiography), surgery for ischemic heart disease, transient ischemic attack (TIA), carotid endarterectomy, peripheral arterial reconstructive surgery, or limb amputation.

All the patients were followed up until the occurrence of an endpoint event, 31 January 2022, or loss of follow-up, whichever came first.

The study was performed in accordance with the Helsinki Declaration and approved by the Clinical Research Ethics Committees of Peking University First Hospital (No. 2016-083). The entire research scheme was explained in detail to each participant, and informed consent was obtained.

### Clinical assessment at baseline

A structured series of clinical assessments were performed for all the enrolled patients at the baseline visit, including demographics, gout history, comorbidities, smoking status, alcohol consumption, blood pressure, treatment, and laboratory tests, for instance, the concentrations of fasting blood glucose, SUA, urea, creatinine, total cholesterol, high-density lipoprotein cholesterol (HDL-C), low-density lipoprotein cholesterol (LDL-C), and triglycerides. They were performed using the standard protocols at Peking University First Hospital by an auto-analyzer (Beckman Coulter AU 680 Chemistry Analyzer, Tokyo, Japan).

Smoking status was self-reported by patients, and those with a lifetime consumption of more than 100 cigarettes were defined as smokers (13). Alcohol consumption was defined as an intake of more than or equal to 100 ml/day for over 1 year. Hypertension was defined when a patient had a history of hypertension (blood pressure ≥ 140/90 mmHg) or was currently taking antihypertensive medications continuously (14). Diabetes mellitus was diagnosed if the subject had a history of diabetes or was currently using antidiabetic drugs (15). Dyslipidemia was defined as a history of dyslipidemia satisfying any one of the following: total cholesterol, ≥ 6.2 mmol/L; triglycerides, ≥ 1.7 mol/L; LDL-C, ≥ 4.14 mmol/L; or HDL-C, <1.0 mmol/L, or currently using lipid-lowering drugs (16). The estimated glomerular filtration rate was calculated using the Chronic Kidney Disease Epidemiology Collaboration creatinine equation (17).

### Ultrasound assessments at baseline and definitions

An ultrasound assessment for joints and carotid arteries was conducted for each patient at baseline. All the ultrasound scanning was performed by two experienced rheumatologists endorsed by EULAR with over 10 years of experience. They were blinded to any clinical and laboratory data about the patients. A GE LOGIQ E9 (GE Healthcare, Waukesha, WI, USA) machine was used for all the scanning.

A musculoskeletal ultrasound assessment was performed for bilateral feet and ankles. Linear transducers, either with ML of 15–6 MHz or a small footprint array of 18–8 MHz were adopted. The scan procedures were based on standardized guidelines published by the Outcome Measures in Rheumatology Clinical Trials (OMERACT) task force (18). The domains for MSU crystal depositions include tophi, double contour sign, and aggregates. The articular inflammatory signs were indicated by the presence of a power Doppler (PD) signal locally.

Ultrasound assessment for bilateral carotid arteries was performed following the Mannheim consensus (19), using a semi-automatic reading system (artery management system) with an 11-MHz linear vascular probe. Six carotid arterial segments were assessed to detect atheroma plaques and measure the intima-media thickness. Carotid plaque (CP) was defined as a cIMT of >1.5 mm or a focal narrowing of ≥0.5 mm of the surrounding lumen, or >50% of the surrounding carotid IMT value (19). Subclinical atherosclerosis was defined when CP was present. A patient with either the presence of CP or cIMT of >0.9 mm was considered to have a high risk of CVD.

To ensure intra- and interobserver reliability, two rheumatologists performed ultrasound assessment on five patients on the same day and repeated the examinations 2 weeks later. The Cohen’s κ values for the intraobserver agreement were 0.76 for the double-contour sign (*p* < 0.0001), 0.70 for tophi (*p* < 0.0001), and 0.60 for aggregates (*p* < 0.0005), respectively. The mean κ values were 0.65–0.75 for the intraobserver agreement (*p* < 0.001) and 0.70 for the interobserver concordance (*p* < 0.0001). These indicated a good level of agreement and reliability.

### Assessment of cardiovascular risk at baseline

The FRS, the most widely used tool for estimating the 10-year CV risk was calculated according to the Framingham Heart Study (20). SCORE2, developed by the European Society of Cardiology, was calculated (21), and QRISK3 was calculated using by the QRISK3-2018 risk calculator with an adjustment for rheumatic diseases (22). These three scores representing the CV risk were evaluated in each patient at baseline. A patient with FRS of >10%, SCORE2 of >5%, and QRISK3 of >20% was considered to have an elevated risk of CVD.

### Study outcomes and definitions

The occurrence of the first MACE during the follow-up was defined as the outcome of the study, including ischemic heart disease, nonfatal myocardial infarction, TIA, stroke, and death due to any of the above reasons (11). Myocardial infarction included ST-segment elevation or non-ST-segment elevation myocardial infarction. Cerebrovascular accidents included ischemic stroke and TIA. Cardiovascular death included sudden cardiac death or death from myocardial infarction, heart failure, cerebrovascular accident, a cardiovascular procedure, or other cardiovascular causes. MACE was adjudicated by two researchers independently.

### Statistical analysis

The mean ± standard deviation (SD) and median (interquartile range) were used to prescribe normally and non-normally distributed data, respectively. Student’s *t*-test and Chi-squared (*χ* ^2^) test were conducted to compare the means and proportions of each group. A Cox proportional hazard regression was used to investigate the baseline CV risk scores, cIMT, CP, and the time to the first MACE occurrence. The effect of the presence of crystal deposition and CP at baseline on MACE-free survival was assessed using Kaplan–Meier survival analysis. The survival distributions were compared by using log-rank testing. Statistical analyses were performed with SPSS for Windows, v. 26.0 (SPSS Inc., Chicago, IL, USA). *p*-values of <0.05 were considered significant. *p*-values of <0.01 were considered highly significant.

## Results

### Patient characteristics and CVD risks at baseline

**Table 1** summarizes the baseline characteristics of 240 gout patients. The average age was 43.3 ± 12.1 years, with a male predominance (238, 99.2%). At baseline, the median disease duration was 3.0 (IQR, 1.0–6.0) years. There were 219 (91.3%) patients having traditional cardiovascular risk factors, with 87 (36.3%), 64 (26.7%), 44 (18.3%), and 24 (10.1%) having with 1, 2, 3, and >3 risk factors, respectively; 99/240 (41.3%) patients were classified as having elevated risk for CV events defined by FRS >10%, 25/240 (10.4%) by SCORE2, and 11/240 (4.6%) by QRISK3, respectively.

**Table 1:** Baseline characteristics of the entire cohort in patients with both carotid and bilateral foot and ankle ultrasound imaging.

Parameters	*n* = 240	*n* = 198 without diabetes and CKD	*p*-value
Age (years)	43.3 ± 12.1	42.1 ± 11.6	0.58
Men (*n* (%))	238 (99.2)	197 (99.5)	0.85
Height (cm)	174.7 ± 5.9	174.2 ± 5.2	0.81
Weight (kg)	80.9 ± 14.9	80.7 ± 14.2	0.46
Presence of conventional CVD risk factors			
BMI (kg/m^2^)	26.6 ± 3.6	26.8 ± 3.8	0.78
Hypertension (*n* (%))	88 (36.7)	62 (31.3)	0.72
Diabetes mellitus (*n* (%))	23(9.6)	0	–
Dyslipidemia (*n* (%))	94 (39.2)	79 (39.9)	0.56
Smoker (*n* (%))	66 (27.5)	55 (27.8)	0.62
Gout-related disease characteristics			
Disease duration in months	47.7 ± 25.4	47.2 ± 25.9	0.93
Number of involved joints	3 (0–4)	3 (0–4)	0.82
Number of flares last year	4 (2–13)	4 (2–13)	0.94
Presence of tophi	112 (46.7)	98 (49.5)	0.78
Patient global (0–100)	40 ± 23	40 ± 21	0.85
Physician global (0–100)	40 ± 21	40 ± 20	0.87
Alcohol intake (*n* (%))	95 (39.6)	84 (42.4)	0.63
**Serum uric acid (µmol/L)**	482.5 ± 130.7	502.3 ± 126.3	0.81
**Creatinine (µmol/L)**	95.3 ± 13.6	93.6 ± 13.5	0.75
**eGFR (ml/min)**	86.8 ± 15.5	87.7 ± 14.7	0.69
Chronic kidney disease			
eGFR <60 ml/min (*n* (%))	23 (9.6)	0	–
eGFR <30 ml/min (*n* (%))	1 (0.4)	0	–
**Fasting glucose (mmol/L)**	5.4 ± 0.3	5.4 ± 0.9	0.47
**Cholesterol (mmol/L)**	4.9 ± 1.0	4.9 ± 1.0	0.46
**HDL (mmol/L)**	1.1 ± 0.2	1.1 ± 0.2	0.39
**LDL (mmol/L)**	3.4 ± 0.5	3.1 ± 0.6	0.82
**Triglycerides (mmol/L)**	2.1 ± 1.0	2.1 ± 1.0	0.95
**HCY (µmol/L)**	5.0 ± 0.5	5.0 ± 0.6	0.82
**hsCRP (mg/L)**	6.3 ± 2.8	6.2 ± 2.7	0.73
Cardiovascular risk scores			
FRS	10.6 ± 8.7	10.2 ± 8.5	0.76
SCORE2	2.8 ± 2.3	1.9 ± 1.0	0.85
QRISK3	1.9 ± 1.2	1.8 ± 1.2	0.78
Treatment at baseline			
Diuretics			
Current (*n* (%))	0 (0)	0 (0)	–
Past (*n* (%))	2 (0.8)	0 (0)	–
Never (*n* (%))	238 (99.2)	198 (100)	0.86
Statins			
Current (*n* (%))	18 (7.5)	9 (4.5)	0.65
Past (*n* (%))	6 (2.5)	1 (0.5)	0.14
Never (*n* (%))	216 (90.0)	188 (95.0)	0.46
Antiplatelet drugs			
Current (*n* (%))	0 (0.0)	0 (0.0)	–
Past (*n* (%))	2 (0.8)	2 (1.0)	0.45
Never (*n* (%))	238 (99.2)	196 (99.0)	–
Urate-lowering therapy			
Current (*n* (%))	0 (0.0)	0 (0.0)	–
Past (*n* (%))	2 (0.8)	2 (1.0)	0.53
Never (*n* (%))	238 (99.2)	196 (99.0)	–
NSAIDs			
Current (*n* (%))	8 (3.3)	7 (3.5)	0.32
Past (*n* (%))	28 (11.7)	20 (10.1)	0.54
Never (*n* (%))	204 (85.0)	171 (86.4)	0.62
Colchicine			
Current (*n* (%))	8 (3.3)	7 (3.5)	0.34
Past (*n* (%))	2 (0.9)	1 (0.5)	0.56
Never (*n* (%))	230 (95.8)	190 (96.0)	0.63
Corticosteroids			
Current (*n* (%))	2 (0.9)	1 (0.5)	0.71
Past (*n* (%))	0 (0.0)	0 (0.0)	–
Never (*n* (%))	238 (99.1)	197 (99.5)	0.62
Carotid ultrasound findings			
Carotid plaque (*n* (%))	45(18.8)	32 (16.2)	0.57
TPA (cm^2^)	64.2 ± 24.4	68.2 ± 28.8	0.72
Increased cIMT (*n* (%))	27 (11.3)	19 (9.6)	0.58
cIMT (mm)	0.91 ± 1.02	0.90 ± 1.11	0.69
Joint ultrasound parameters			
Positive PD signal (≥1)	55 (22.9)	49 (24.7)	0.64
Tophi (≥2)	16 (6.7)	11 (5.6)	0.87
Double-contour sign	83 (34.6)	70 (35.4)	0.75
Aggregates	22 (9.2)	18 (9.1)	0.69
Erosion	53 (22.1)	43 (21.7)	0.77

CVD, cardiovascular disease; BMI, body mass index; PD, power Doppler; eGFR, estimated glomerular filtration rate; HDL, high-density lipoprotein; LDL, low-density lipoprotein; HCY, homocysteine; hsCRP, highly sensitive C-reactive protein; FRS, Framingham risk score; NSAIDs, nonsteroidal anti-inflammatory drugs; IMT, intima-media thickness; TPA, total plaque area; CIMT, carotid intima-media thickness; N/A, not available.

For diabetes and chronic kidney disease to favor the development of atherosclerosis and cardiovascular disease, patients with diabetes or chronic kidney disease (eGFR level below 60 ml/min/1.73 m^2^) were compared with the entire cohort. There was no significant difference in clinical and laboratory parameters between the diabetes and chronic kidney disease group and the entire group at baseline (**Table 1**).

### Ultrasound findings at carotid arteries at baseline

Regarding the carotid scans, atheroma plaques were present in 45 individuals (18.8%) and were found bilaterally in 24 (10.0%). The mean TPA (total plaque area) was 64.2 ± 24.4 cm^2^. Twenty-seven (11.3%) participants showed increased IMT. The mean cIMT was 0.91 ± 1.02 mm.

### Musculoskeletal ultrasound findings at baseline

The typical crystal deposition was found by ultrasound in 143 (59.6%) patients, presenting as tophi in 132 (55.0%) patients, DCS (double contour sign) in 83 (34.6%) patients, and aggregates in 22 (9.2%) patients. PD signal was observed in 22.9% (55/240) and bone erosions in 22.1% (53/240) patients, all in MTP1 joints. Among the 143 patients, the crystal deposition was most commonly located in MTP1 (first metatarsophalangeal) joint (87, 60.8%), Achilles tendon (49, 34.3%), ankle joint (7, 5.0%), and other regions. Among the 132 patients with tophi, four (3.0%) had four tophi, 12 (9.1%) had two tophi, and 116 (87.9%) had one tophus in the bilateral foot and ankle region.

### MACE during follow-up

A total of 2,441 patient-years of follow-up were available for analysis. The median follow-up period was 10.3 (6.0–14.0) years, and 187 (77.9%) patients were followed up for more than 10 years. During follow-up, 28 (11.7%) patients experienced MACE (1.1 events per 100 patient-years), with stroke (18, 7.5%), acute myocardial infarction (six, 2.5%), TIA (five, 2.1%), and acute myocardial infarction along with stroke (one, 0.4%).

Compared to 212 MACE-free patients, those experiencing MACE during follow-up were older, more likely to have hypertension, diabetes, dyslipidemia, and higher FRS and QRISK3 scores at baseline. Moreover, the patients with MACE had a longer duration of gout, more joint involvement, and more tophi on ultrasound (**Table 2**).

**Table 2:** Clinical characteristics of patients with and without MACE in the entire cohort (*n* = 240).

	Entire cohort (*n* = 240; median (IQR) or mean ± SD or *n* (%))		
	MACE− (*n* = 212)	MACE+ (*n* = 28)	*p*-value
Clinical demographic parameters			
** Age (years)**	39.3 ± 11.5	44.6 ± 12.1	0.029^*^
** Men (*n* (%))**	210 (99.0)	28 (100)	0.606
** Gout duration (years)**	3.0 (1.0, 6.0)	5.5 (2.0, 8.0)	0.009^*^
Cardiovascular risk factors			
** BMI (kg/m^2^)**	26.4 ± 3.8	27.1 ± 2.1	0.131
** Hypertension (*n* (%))**	66 (31.1)	22 (78.6)	0.001^*^
** Diabetes (*n* (%))**	17 (8.0)	6 (21.4)	0.050^*^
** Dyslipidemia (*n* (%))**	74 (34.9)	20 (71.4)	0.001^*^
** Smoker (*n* (%))**	51 (24.1)	15 (53.6)	0.001^*^
Gout-related disease characteristics			
** Number of involved joints**	1 (1, 2)	1 (1, 3)	0.621
** Number of flares last year**	4.0 (2.0, 12.0)	10.0 (4.5, 17.5)	0.001^*^
** Presence of tophi**	91 (42.9)	21 (75.0)	0.001^*^
** Patient global (0–100)**	50 (20, 40)	60 (20,50)	0.358
** Physician global (0–100)**	20 (10, 30)	20 (10, 30)	0.309
** Alcohol intake (*n* (%))**	75 (35.4)	20 (71.4)	0.001^*^
** Fasting glucose (mmol/L)**	5.5 ± 0.8	6.3 ± 1.2	0.050^*^
** Serum uric acid (µmol/L)**	489.2 ± 130.8	568.8 ± 96.7	0.002^*^
** Creatine (µmol/L)**	96.4 ± 15.1	122.5 ± 12.6	0.151
** eGFR (ml/min)**	85.1 ± 16.3	79.5 ± 12.1	0.245
Chronic kidney disease			
** GFR <60 ml/min (*n* (%))**	19 (9.0)	4 (14.3)	0.368
** GFR <30 ml/min (*n* (%))**	0 (0.0)	1 (3.6)	0.006^*^
** Cholesterol (mmol/L)**	4.8 ± 1.0	4.7 ± 1.1	0.491
** HDL (mmol/L)**	1.1 ± 0.2	0.8 ± 0.2	0.001^*^
** LDL (mmol/L)**	2.7 ± 0.7	3.1 ± 0.8	0.038^*^
** Triglycerides (mmol/L)**	2.1 ± 1.0	2.7 ± 0.9	0.132
** HCY (µmol/L)**	18.4 ± 6.8	20.5 ± 13.9	0.314
** hsCRP**	4.1 ± 2.8	8.9 ± 2.4	0.289
Cardiovascular risk scores			
** FRS**	10.5 ± 8.3	15.0 ± 11.0	0.040^*^
** SCORE2**	2.8 ± 2.6	2.6 ± 2.9	0.792
** QRISK3**	1.6 ± 1.0	4.2 ± 1.3	0.001^*^
Current treatment at baseline			
** NSAIDs (*n* (%))**	5 (2.4)	3 (10.7)	0.053
** Steroid (*n* (%))**	1 (0.5)	1 (3.5)	0.312
** Statin (*n* (%))**	17 (8.0)	1 (3.6)	0.401
Carotid ultrasound parameters			
Carotid plaque (*n* (%))	27 (12.7)	18 (64.3)	0.001^*^
Mean carotid IMT (mm)	0.8 ± 0.2	1.4 ± 0.4	0.01^*^
Maximum carotid IMT (mm)	0.9 ± 0.3	1.6 ± 0.5	0.01^*^
Increased cIMT (*n* (%))	85 (40.1)	17 (60.7)	0.001^*^
TPA (cm^2^)	20.6 ± 3.7	131.9 ± 85.8	0.03^*^
Joint ultrasound parameters			
**Positive PD signal (≥1)**	52 (24.5)	3 (10.7)	0.102
**Tophi (≥2)**	4 (1.9)	12 (42.9)	0.001^*^
**Double-contour sign**	69 (32.5)	14 (50.0)	0.068
**Aggregates**	19 (9.0)	3 (10.7)	0.651
**Erosion**	43 (20.3)	10 (35.7)	0.060

^*^Significant at p ≤ 0.05.

BMI, body mass index; eGFR, estimated glomerular filtration rate; hsCRP, highly sensitive C-reactive protein; HCY, homocysteine; MACE+, patients who developed cardiovascular event; MACE−, patients who did not develop cardiovascular event; FRS, Framingham risk score; SCORE2, Systematic Coronary Risk Evaluation 2; HDL, high-density lipoprotein; LDL, low-density lipoprotein; IMT, intima-media thickness; TPA, total plaque area; N/A, not available; NSAIDs, nonsteroidal anti-inflammatory drugs; PD, power Doppler.

### The predictors for MACE in gout patients

Among 28 patients who experienced MACE, there were 14 (50.0%), two (7.1%), and 23 (82.1%) patients identified as having elevated CV risk, respectively, defined by FRS, SCORE2, and QRISK3 at baseline.

The association of MACE occurrence with gouty arthritis, such as the number of flares from baseline in the previous, the presence of tophi, the double-contour sign, and the PD signal on ultrasound, is shown in **Table 3**. MACE was significantly more common in patients with tophi than those without by physical examination (91/212 (42.9%) vs. 21/28 (75.0%); *p* = 0.001), in patients with at least two tophi than less than two tophi on ultrasound (4/212 (1.9%) vs. 12/28 (42.9%); *p* = 0.001), and in patients with CP than those without CP (27/212 (12.7%) vs. 18/28 (64.3%); *p* = 0.001).

**Table 3:** Univariate and multivariate Cox regression analyses for parameters as predictors for MACE (*n* = 240).

	Univariate analysis		Multivariate analysis	
	HR (95% CI)	*p*-value	HR (95% CI)	*p*-value
**Age**	1.03 (1.01, 1.23)	0.321		
**Disease duration**	1.23 (1.03, 1.42)	0.587		
**Hypertension**	2.14 (1.42, 5.18)	0.002^*^		
**Alcohol intake**	1.92 (1.72, 3.32)	0.002^*^		
**eGFR <60ml/min**	2.58 (0.89, 7.52)	0.083		
**Diabetes**	1.42 (1.24, 1.72)	0.001^*^		
**Dyslipidemia**	1.11 (1.02, 2.56)	0.459		
**Smoker**	2.18 (1.43, 4.93)	0.003^*^		
**Number of flares last year**	1.05 (1.02, 1.09)	0.004^*^		
**Patient global (0–100)**	1.01 (1.00, 1.04)	0.123		
**Physician global (0–100)**	1.00 (0.99, 1.01)	0.871		
**Presence of tophi**	3.15 (1.34, 7.42)	0.009^*^		
**Positive PD signal (≥1)**	0.42 (0.13, 1.38)	0.152		
**Tophi (≥2)**	7.53 (7.92, 38.8)	0.001^*^	9.63 (6.12, 39.8)	0.001^*^
**Double-contour sign**	1.74 (0.81, 3.72)	0.154		

^*^Significant at p ≤ 0.05.

Univariable Cox regression analysis revealed that a higher number of gout flares in the previous year before enrollment (HR, 1.05 (95% CI, 1.02–1.09); *p* < 0.05), presence of at least two tophi (HR, 7.53 (95% CI, 7.92–38.8); *p* = 0.001), carotid plaque (HR, 7.36 (95% CI, 3.36–16.1); *p* = 0.001) on ultrasound were associated with a higher risk of developing MACE. Although in univariate analysis, the variables associated with MACE were hypertension, age, disease duration, diabetes, alcohol intake, and smoking, in the multivariate model, only the presence of at least two tophi was associated with MACE, with an HR of 9.63, and this effect was independent of other variables. There is no significant association of mean carotid IMT or maximum carotid IMT with MACE (*p* = 0.23; *p* = 0.061) (**Table 3**).

In the multivariable model, at least two tophi and carotid plaque were both independent predictors of MACE after adjusting for all CVD risk scores (HR ranged from 2.12 to 5.25 and 3.72 to 4.01; *p* < 0.05) in three models separately (**Table 4**).

**Table 4:** Multivariable Cox regression model for predicting MACE (n=240).

	Model 1^a^		Model 2^b^		Model 3^c^	
	HR (95% CI)	*p*-value	HR (95% CI)	*p*-value	HR (95% CI)	*p*-value
Carotid plaque	3.72 (1.26–10.90)	0.017^*^	4.01 (1.64–10.30)	0.001^*^	3.92 (1.29–10.10)	0.011^*^
Tophi (≥2)	5.25 (2.55–26.72)	0.001^*^	3.42 (3.23–7.13)	0.003^*^	2.12 (2.11–9.31)	0.050^*^
FRS	1.05 (1.01–1.09)	0.005^*^	–			
SCORE2	–		2.15 (1.82–4.23)	0.432		
QRISK3	–				2.71 (1.67–2.99)	0.014^*^

^*^Significant at p ≤ 0.05.

a Adjusted for FRS.

b Adjusted for SCORE2.

c Adjusted for QRISK3.

FRS, Framingham risk score; SCORE2, Systematic Coronary Risk Evaluation 2.

**Figure 1:**
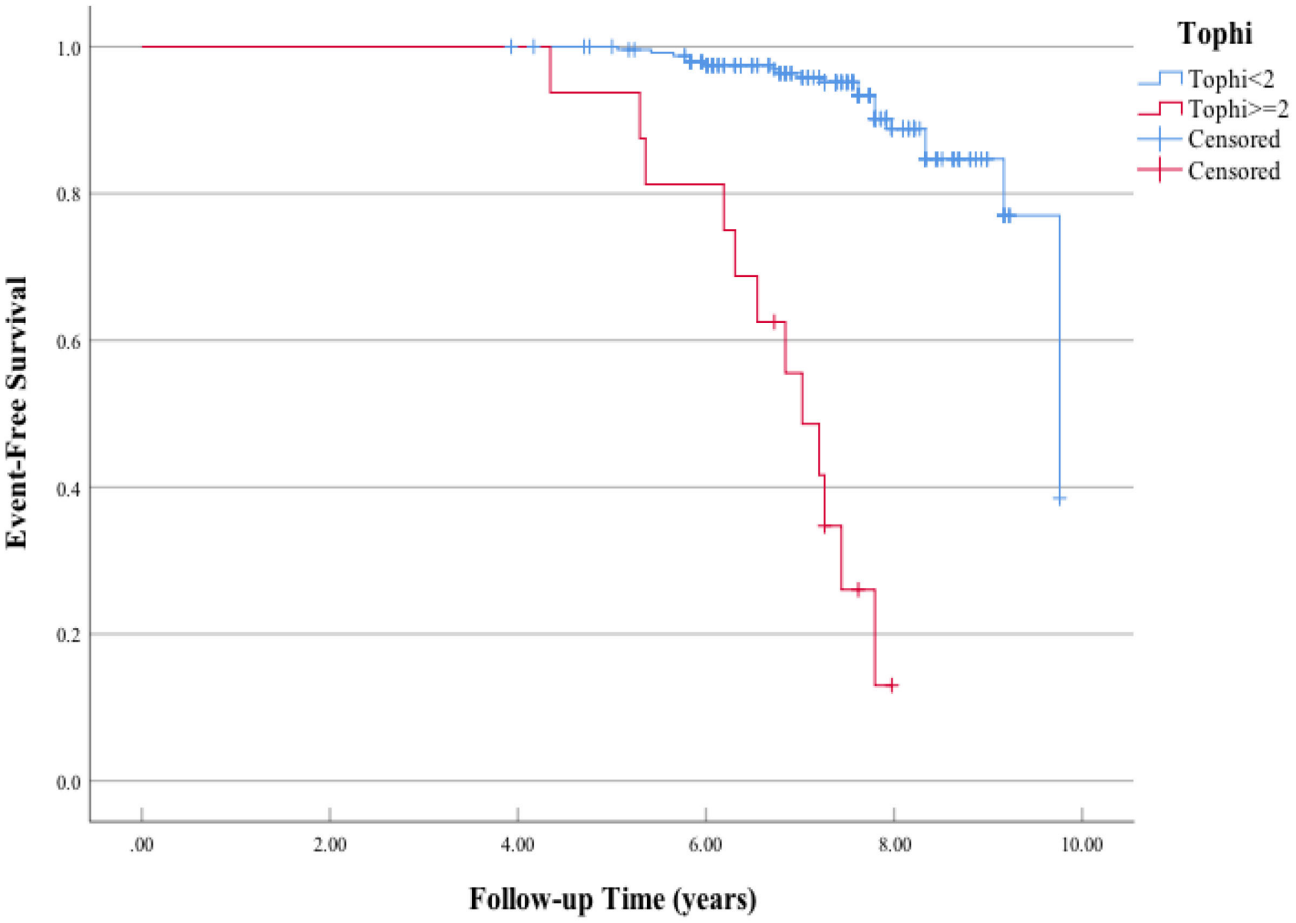
Kaplan–Meier curves of the association between tophi detected by ultrasonography at baseline and major adverse cardiac events (MACE). Log-rank *χ* ^2^ (1 *df*) = 99.8 (*p =* 0.001) for differences in the event-free survival rate between patients who had positive carotid plaque and those who had negative carotid plaque.

**Figure 2:**
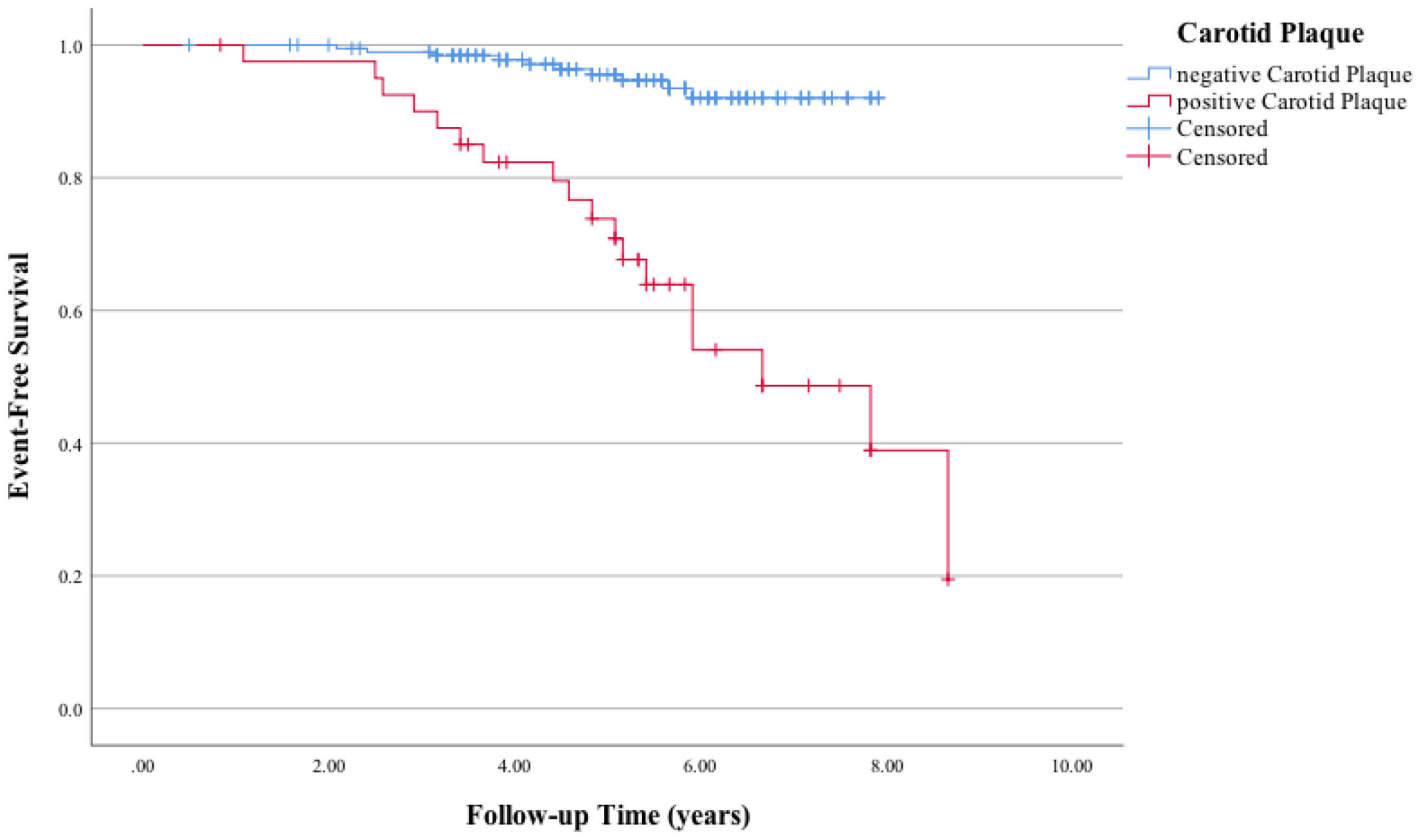
Kaplan–Meier curves of the association between carotid plaque and major adverse cardiac events (MACE). Log-rank *χ* ^2^ (1 *df*) = 36.7 (*p =* 0.001) for differences in the event-free survival rate between patients who had positive carotid plaque and those who had negative carotid plaque.

A significant difference in MACE-free survival was observed between patients with at least two tophi and less than two tophi (**Figure 1**). The Kaplan–Meier survival curve indicated that the MACE-free survival was significantly lower in patients with CP compared with those without (**Figure 2**).

## Discussion

MACE, CVD in particular, has been the leading cause of morbidity and mortality. Prospective studies demonstrating that the predictive CV tools designed for the general population underestimate the CV risk in patients with inflammatory diseases are scarce, and this is the first study confirming this point in gout. Patients with gout have a higher risk of CVD, independent of traditional cardiovascular risk factors (23). In this cohort study, we demonstrate that the baseline disease burden, as reflected by the presence of at least two tophi, is an independent predictive factor for incident MACE after adjusting for traditional cardiovascular risk scores. To the best of our knowledge, this is the first study reporting the association of the presence of at least two tophi and carotid plaque on ultrasound with MACE in addition to traditional cardiovascular risk factors in a long-term follow-up cohort of gout patients. Ultrasound evaluations in joints and carotid arteries are especially useful for detecting subclinical tophi and subclinical arteriosclerosis and significantly improving cardiovascular risk assessment in gout patients. It supports the need to use additional validated tools like carotid US to improve CV estimation in inflammatory conditions in general and in particular in gout; the presence of carotid plaques also allows for the reclassification of several patients into very-high CV risk.

Gout flares are characterized by neutrophil-rich acute inflammation due to NLRP-3 inflammasome activation (24). A recently published study based on a large nationwide database of gout patients suggested that gout flares were associated with a subsequently transient increase in cardiovascular events (25). Our results showed that the frequency of gout flares in the previous year of the baseline was also associated with subsequent MACE. Neutrophilic inflammation has been confirmed to be associated with instability and rupture of atherosclerotic plaque (26). Activated intraplaque inflammatory cells can upregulate the response proteins, including metalloproteinases and peptidases, and further promote oxidative stress, all of which contribute to plaque destabilization (27). This may explain the association of cardiovascular events with prior gout flares. A randomized clinical trial has demonstrated that blocking the NALP3 inflammasome indeed prevented recurrent cardiovascular events (28).

In this study, we found that those traditional cardiovascular risk scores appeared to be suboptimal as risk assessment tools in patients with gout, although the performance of FRS and QIRSK3 was better than the SCORE2. A previous study showed that both FHS and SCORE appeared to underestimate the presence of carotid plaque in newly diagnosed gout patients (5). EULAR recommends screening for CVD risk in all patients with inflammatory arthritis and states that rheumatologists should be responsible for risk evaluation in 2022 (29). Unfortunately, no study has ever investigated the accuracy of these CVD prediction tools in patients with gout (29). Our data showed that FRS and QRISK3 can be used in gout patients in future clinical practice. With respect to this, a recent study proposed the combined use of QRISK3 and EULAR-modified SCORE2 as an alternative to the assessment of carotid plaques by ultrasound to identify patients with rheumatoid arthritis at high risk of cardiovascular disease (30). Therefore, it could be an option to be assessed in patients with gout when an ultrasound study is not available.

Clinically detectable tophi have been confirmed to be predictive of long-term MACE. Importantly, our study for the first time revealed that an equal or more than two tophi shown on ultrasound can also predict long-term MACE in gout patients. The number of tophi counted *via* ultrasound was the key predictive factor for the incident MACE as a comorbidity of gout. This provides a new understanding of the role of MSU crystal burden in cardiovascular risk. Similar to our results, the volume of MSU crystals measured by dual-energy computed tomography has also been identified as a biomarker for the risk of developing new cardiometabolic diseases and all-cause mortality (31). Persistent deposition of MSU crystals can induce chronic inflammation (32). Controlling the chronic subclinical inflammation might improve cardiovascular mortality with medications, such as colchicine (33) and canakinumab (28). MSU crystallization can form by templated nucleation, and inflammatory cells rapidly react to the subclinical tophi (34). Ultrasound is definitely more sensitive than physical examination in detecting subcutaneous tophi, especially in joints (35). The exuberant inflammation surrounding large crystals in tophi was the largest source of chronic inflammation and may explain the link between gout and increased cardiovascular risk. Our study suggests that the overall crystal burden, including the subclinical tophi detected by ultrasonography, has a great impact on chronic inflammation and drives most of the MACE in gout. An observational study investigated specimens of coronary arteries from an explanted heart by polarization microscopy and found urate crystal deposition in about 10% of coronary arteries (36). Whether crystal deposition in the vessels contributes to a higher cardiovascular risk needs to be verified in the future.

In the analysis, we also found that ultrasound measurements of carotid atherosclerosis, as presented by carotid plaque, are an independent risk factor for MACE in gout. The presence of carotid plaque was associated with an approximately threefold increase in the risk of developing incident MACE after adjusting for traditional cardiovascular risks, which is comparable with that reported in patients with RA (37) or PsA (38). A prospective 5-year follow-up study confirmed that the presence of carotid plaques predicted the development of cardiovascular events and death in patients with RA (39). A recent study showed that the presence of tophi and a positive power Doppler signal were significantly associated with atheroma plaques in 103 Spanish gout patients (40). Also, the increased IMT measurement showed no association with MSU crystal, which is similar to our study. There is no correlation between the percentage of positive PD signals and MACE in our study. Differences in ethnicity, the severity of gout such as young age, a lower percentage of conventional CVD risk factors (such as diabetes and hypertension), as well as the acute or intercritical phase to acquire the inflammation in the US can be confounding factors. Moreover, hsCRP at baseline is not a risk factor or a PD signal in our study. Previous data remind us that baseline CRP level is a predictor of CV mortality and CV events in rheumatoid arthritis patients (41). Further study showed that while a single isolated determination of CRP may not be associated with subclinical atherosclerosis (increased carotid intima-media thickness) in patients with rheumatoid arthritis (42), the average CRP value over an extended long period of follow-up may reflect the chronicity of the inflammatory. Both tophi and carotid plaque were accumulated effects of gout; data from the cross-sectional study at baseline or follow-up points cannot explain the incidence of MACE.

The underlying pathophysiological mechanisms, such as systemic inflammation, elevated oxidative stress, endothelial dysfunction, and changes in lipid profiles, might contribute to a higher CVD risk in gout patients. Two retrospective cohort studies suggested a protective effect of statins on mortality in patients with gout compared to those without statins (43, 44). In our cohort, we did not find a relationship between statin use and MACE.

There are several strengths of the present study. First, this is the first prospective study to investigate the added value of MSU crystal deposition and abnormal carotid manifestations for predicting incidents of MACE in subclinical CVD gout patients. In the study, ultrasound assessment of the carotid arteries and joints enabled us to compare the subclinical characteristics. Second, the follow-up period was long in the study, with a median of 10.3 (6.0–14.0) years and more than 10 years in 187 (71.3%) patients. Events can occur late, and the long-term follow-up and low loss of data guarantee this dataset may be best available in the future. Third, the analyses were adjusted for widely used CVD risk predictive tools. This supports the notion that subclinical atherosclerosis is on the causal pathway rather than a confounder. As the Chinese population has a lower CVD risk in general (45), the European low-risk chart was adopted using SCORE2. Most of the previous studies did not adjust the analysis to determine the predictive value of their findings compared to existing clinical tools. As this is a middle-aged population without a high degree of established comorbidity, the new incidence of MACE prompts the importance of detecting tophi and carotid plaque by ultrasonography in daily clinical practice.

We acknowledge some limitations. First, MACE as the primary outcome occurred in a limited number of patients. There were only 28 patients who developed incident MACE, but this has already been the only longitudinal study with a 10-year follow-up. Future studies with longer follow-ups are warranted. Second, this was a single-center study, and the results may be not generalized to other populations. Third, the CV risk algorithms calculated in this study have not been designed for Asian populations. Last, since the follow-up period was long, we were not able to list and discuss the choice and effect of urate-lowering therapy.

In conclusion, ultrasonography can detect atherosclerosis and crystal deposits in joints and surrounding tissues. This study shows the presence of at least two tophi and carotid plaque on ultrasound, which independently predicted incident MACE in addition to conventional cardiovascular risk factors. Combining ultrasound assessment on carotid arteries can improve the accuracy of cardiovascular risk based on traditional cardiovascular risk factors in patients with gout.

## Data availability statement

The raw data supporting the conclusions of this article will be made available by the authors, without undue reservation.

## Ethics statement

The studies involving human participants were reviewed and approved by Clinical Research Ethics Committees of Peking University First Hospital. Written informed consent for participation was not required for this study in accordance with the national legislation and the institutional requirements.

## Author contributions

All authors had access to the data and played a role in writing the manuscript. ZZ designed the research protocol and critically revised the manuscript. XD and XZ extracted the ultrasound data. YW designed the research protocol, had full access to all data collection, performed the statistical analysis, and wrote the manuscript. All authors contributed to the article and approved the submitted version.
