# Supplementary material for: Presence of tophi and carotid plaque were risk factors of MACE in subclinical artherosclerosis patients with gout: a longitudinal cohort study
Source: Front Immunol. 2023 Apr 18;14:1151782. doi: 10.3389/fimmu.2023.1151782 (PMC10153647; doi:10.3389/fimmu.2023.1151782)
Supplement: Supplementary file 1 [file Table_1.docx]

Supplemental Table 1

| **Parameters** | n=240 | n=112  with tophi | n=128  without tophi | *P* |
| --- | --- | --- | --- | --- |
| Age (years) | 43.3±12.1 | 41.3±11.5 | 45.0±12.4 | 0.02* |
| Male, n (%) | 238 (99.2) | 111 (99.1) | 127 (99.2) | 0.85 |
| Height (cm)  Weight (kg)  **Conventional CVD risk factors present**  BMI (kg/m^2^)  Hypertension, n (%)  Diabetes mellitus, n (%)  Dyslipidemia, n (%)  Smoker, n (%) | 174.7±5.9  80.9±14.9  26.6±3.6  88 (36.7)  23(9.6)  94 (39.2)  66 (27.5) | 173.9±5.1  81.7±15.2  26.9±3.8  53 (47.3)  7 (6.3)  45 (40.2)  38 (33.9) | 175.2±4.9  80.2±13.4  26.3±3.6  35 (27.3)  16 (12.5)  49 (38.3)  28 (21.9) | 0.81  0.46  0.22  0.01*  0.13  0.76  0.06 |
| **Gout-related disease characteristics** |  |  |  |  |
| Disease duration in months  Number of involved joints  Number of flares last year  Patient global (0-100)  Physician global (0-100)  Alcohol intake, n (%) | 47.7±25.4  3 (0-4)  4 (2-13)  40±23  40±21  95 (39.6) | 53.3±23.9  3 (0-4)  4 (2-13)  40±21  40±19  53 (47.3) | 41.5±25.9  2 (0-4)  3 (2-10)  40±11  40±20  42 (32.8) | 0.73  0.85  0.74  0.65  0.87  0.03* |
| **Serum uric acid (umol/L)**  **Creatine (umol/L)**  **eGFR, ml/min**  **Chronic kidney disease**  eGFR<60ml/min, n (%)  eGFR<30ml/min, n (%)  **Fasting glucose (mmol/L)**  **cholesterol (mmol/L)**  **HDL (mmol/L)**  **LDL (mmol/L)**  **triglycerides (mmol/L)**  **HCY (umol/L)**  **hsCRP (mg/L)**  **Cardiovascular risk scores**  FRS  SCORE2  QRISK3  **Treatment at baseline**  Statins  Current, n (%)  Past, n (%)  Never, n (%)  Antiplatelet drugs  Current, n (%)  Past, n (%)  Never, n (%)  Urate-lowering therapy  Current, n (%)  Past, n (%)  Never, n (%)  NSAIDs  Current, n (%)  Past, n (%)  Never, n (%)  Colchicine  Current, n (%)  Past, n (%)  Never, n (%)  Corticosteroids  Current, n (%)  Past, n (%)  Never, n (%)  **Carotid ultrasound findings**  Carotid plaque, n (%)  TPA, cm^2^  Increased cIMT, n (%)  cIMT, mm  **Joint ultrasound parameters**  Positive PD signal (≥1)  Double contour sign  Aggregates  Erosion | 482.5±130.7  95.3±13.6  86.8±15.5  23 (9.6)  1(0.4)  5.4±0.3  4.9±1.0  1.1±0.2  3.4±0.5  2.1±1.0  5.0±0.5  6.3±2.8  10.6±8.7  2.8±2.3  1.9±1.2  18 (7.5)  6 (2.5)  216 (90.0)  0 (0.0)  2 (0.8)  238 (99.2)  0 (0.0)  2 (0.8)  238 (99.2)  8 (3.3)  28 (11.7)  204 (85.0)  8 (3.3)  2 (0.9)  230 (95.8)  2 (0.9)  0 (0.0)  238 (99.1)  45(18.8)  64.2±24.4  27(11.3)  0.91±1.02  55 (22.9)  83 (34.6)  22 (9.2)  53 (22.1) | 512.4±131.8  100.4±34.9  86.5±16.7  9 (8.0)  1 (0.8)  5.7±1.2  4.9±1.0  1.0±0.2  3.1±0.8  2.2±1.2  4.8±0.6  6.2±2.1  10.5±8.7  3.1±2.8  2.1±1.5  9 (8.0)  4 (3.6)  99 (88.4)  1 (0.9)  111 (99.1)  1 (0.9)  111 (99.1)  6 (5.3)  20 (17.9)  86 (76.8)  4 (3.4)  1 (0.8)  107 (90.6)  2 (1.8)  110 (98.2)  35 (31.3)  41.4±28.4  20 (17.9)  0.99±1.12  29 (25.9)  54 (48.2)  10 (8.9)  30 (26.8) | 489.1±129.3  98.3±36.6  82.3±17.7  14 (10.9)  0  5.6±0.7  4.7±1.0  1.1±0.2  3.0±0.8  2.1±1.1  4.2±0.4  5.9±2.1  10.8±8.8  2.6±2.3  1.8±1.1  9 (7.0)  2 (1.6)  117 (91.4)  1 (0.8)  127 (99.2)  1 (1.0)  127 (99.2)  2 (1.5)  8 (6.3)  118 (92.2)  4 (3.1)  1 (0.8)  123 (96.1)  0 (0.0)  128 (99.5)  22 (17.2)  34.2±28.8  7 (5.5)  0.83±0.88  26 (20.3)  29 (22.7)  12 (9.4)  23 (18.0) | 0.18  0.65  0.07  0.51  -  0.23  0.19  0.06  0.23  0.79  0.82  0.73  0.74  0.19  0.06  0.56  0.66  0.32  0.67  0.73  -  0.67  0.73  0.43  0.54  0.64  0.52  0.61  0.43  -  -  -  0.81  0.01*  0.34  0.02*  0.35  0.01*  0.91  0.12 |

*Significant at p≤0.05.
